# Trajectories of satisfaction with work–family reconciliation among midlife employees: the role of family-related factors and quality of life

**DOI:** 10.1093/eurpub/ckae117

**Published:** 2024-07-25

**Authors:** Subas Neupane, Tea Lallukka, Aino Salonsalmi, Eija Haukka, Päivi Leino-Arjas

**Affiliations:** Unit of Health Sciences, Faculty of Social Sciences, Tampere University, Tampere, Finland; Gerontology Research Center, Tampere University, Tampere, Finland; Centre of work ability and work careers, Finnish Institute of Occupational Health, Helsinki, Finland; Department of Public Health, University of Helsinki, Helsinki, Finland; Department of Public Health, University of Helsinki, Helsinki, Finland; Centre of work ability and work careers, Finnish Institute of Occupational Health, Helsinki, Finland; Centre of work ability and work careers, Finnish Institute of Occupational Health, Helsinki, Finland

## Abstract

We studied the developmental trajectories of satisfaction with work–family reconciliation (WFS) and their associations with family—related factors and quality of life measures among municipal employees. The study was based on the Helsinki Health Study of municipal employees of the City of Helsinki in 2001–02 and its follow-up surveys in 2007, 2012, and 2017. Employees aged 40–50 at baseline and working at all timepoints were analysed (*n* = 1681, 84% women). Growth Mixture Models were applied to identify trajectories of WFS (dissatisfied vs. satisfied). Associations of family-related and quality-of-life factors (physical functioning and emotional well-being) with the WFS trajectories were studied using log-binomial regression models, adjusting for sociodemographic and lifestyle variables. Two WFS trajectories, low (women 45%; men 53%) and high were identified. In a fully adjusted model among women, having ≥1 children aged 0–6 years was associated with increased odds of belonging to the low WFS trajectory (OR 1.52, 95% CI 1.19–1.95). Among men, having ≥1 children aged 7–18 was associated with decreased odds (0.39, 0.19–0.80). High emotional well-being was inversely associated with the low WFS trajectory among both genders (women 0.32, 0.23–0.45; men 0.20, 0.09-0.46). High physical functioning (0.59, 0.42–0.83) was inversely associated with the low WFS trajectory among women only. Less than half of the women and more than half of the men participants belonged to a low WFS trajectory, which associated with the age of children in the family and quality-of-life measures.

## Introduction

Work–family conflict (WFC), or difficulties reconciling the requirements of paid work with family roles, has grown into a public health concern due to the increased participation of women in the workforce, and has been magnified by the COVID-19 pandemic [1]. WFC is associated with adverse effects on mental [2] and physical health [3, 4], as well as increased absenteeism from work [5, 6].

WFC is assumed to arise when the time or energy demands of one role interfere with those of another, creating stress. This may occur in either direction at the work–family interface: work can affect family life (work-to-family conflict) or vice versa (family-to-work conflict) [7]. However, the measures reflecting the two are rather highly correlated [8]. Both types of conflict are also associated with reduced self-rated health and mental well-being [4, 9] and predict sickness absences particularly due to mental disorders [5]. On the other hand, success in combining work and family roles can provide benefits and opportunities for life enrichment [10]. The use of work–family reconciliation policies such as parental leave and formal childcare are associated with reduced WFC [11].

WFC has been conceptualized and assessed in various ways, using both single- and multi-item instruments. However, review studies [3, 8] have shown that despite heterogeneity, WFC is commonly linked to poorer health. A study based on data from 28 European countries, drawing from the European Working Conditions Survey and using the same method to assess conflict, found that the level of WFC varied relatively little across countries but that women generally experienced somewhat higher WFC than men [12]. The needs of children and informal caregiving commonly increased WFC [12].

The way in which WFC develop over the life course is poorly understood. Earlier longitudinal studies have usually focused on one or both [13] directions of conflict using occupation-specific multi-item measures, often with short follow-up periods [14]. Studies using more general single-item measures, [15, 16] have not explored longitudinal developmental patterns.

A Finnish study of university employees identified four distinct work–family balance profiles: active, passive, beneficial, and detrimental [14] and showed that employees were more likely to align with the beneficial profile if they reported a stronger sense of job control and higher core self-evaluations. Another Finnish study among 277 midlife individuals [17] identified a high mean-level stability of WFC over a 14-year time span, but also four latent trajectories: a decreasing trajectory of work-to-family conflict, a trajectory where both work-to-family and family-to-work conflicts were low, and two very small additional trajectories.

Previous research has shown that the family structure and the degree of responsibility for dependent care are associated with WFC, and that these affect mothers and fathers differently [18]. For instance, mothers may experience higher WFC, and fathers may experience lower WFC. Notably, family-related factors, such as family structure and having dependent children, play a role in an individual’s ability to balance the demands of the work and family domains. Moreover, quality of life, such as physical and emotional well-being, has not been previously explored in relation to the trajectories of WFC.

Based on theories of career development [19], family development [20], and work–family interface [21], it is likely that WFC is more prevalent during early midlife and midlife than late midlife. This suggests that WFC may ease as working parents' children reach adolescence. Therefore, in this study, we aimed to explore the trajectories of satisfaction with work–family reconciliation from midlife to the end of work life separately among women and men, using a large occupational cohort and a long follow-up of 15–17-years. We also examined the role of family-related factors and quality of life in relation to the trajectories.

## Methods

We used the questionnaire data of the prospective Helsinki Health Study [22]. The sample was drawn from the employees of the City of Helsinki, Finland. The baseline survey (Phase 1) was conducted on all employees who reached the age of 40, 45, 50, 55, or 60 in 2000, 2001, or 2002 (*n* = 8960; response rate 67%). Follow-up surveys (Phases 2–4) were conducted in 2007 (*n* = 7332; response rate 83%), 2012 (*n* = 6814, response rate 79%), and 2017 (*n* = 6832, response rate 82%). This study utilized data on the employees who responded to all surveys, were working (either full-time, part-time, or on part-time pension) at all four timepoints and were aged 40–50 in Phase 1 (*n* = 1681, 84% women).

The study was approved by the ethics committee of the Department of Public Health, University of Helsinki, and the health authorities of the City of Helsinki, Finland.

### Satisfaction with work–family reconciliation

Satisfaction with work–family reconciliation (WFS) was assessed at all four timepoints using a single-item question: ‘How satisfied or dissatisfied are you with your work–family reconciliation’? with the seven response options used in Phases 1–3 (1 = very satisfied, satisfied, somewhat satisfied, neither satisfied nor dissatisfied, somewhat dissatisfied, dissatisfied, 7 = very dissatisfied). In Phase 4, the response was measured on a scale of 1–4 (1 = very satisfied, satisfied, dissatisfied, 4 = very dissatisfied). The responses from all surveys were dichotomized (very satisfied or satisfied vs dissatisfied) for the analysis.

### Family-related factors

Each survey inquired whether the respondent lived with a spouse or legal partner (yes/no). The number of (underaged) children (0–6 years) and of children aged 7–18 years living in the same household, were elicited at baseline. In this analysis, we dichotomized the variables as no vs ≥1 for both questions. In the follow-up surveys, we elicited the number of children aged 0–18 years (from 0 to 9 or more children) and dichotomized the responses as no vs one or more children (≥1)

### Quality of life

Physical functioning and emotional well-being were measured as part of RAND-36, a widely used measure of health-related quality of life [23]. The RAND-36 yields eight scale scores and two scales, of which we used physical functioning and emotional well-being measures in this analysis. Each scale was scored on a range of 0–100, higher values indicating better functioning/well-being, and were categorized into three groups, using their tertial values as cut-off points.

### Sociodemographic characteristics

*Age* was categorized as 40, 45, or 50 years, and education as low, intermediate, or high.

*Socioeconomic position* was defined using the occupational titles of the employees and dichotomized as ‘high’ for managers and semi-professionals, and ‘low’ for routine non-manual workers and manual workers [24].

*Body mass index* (BMI; kg/m^2^) was based on self-reported height and weight and classified as normal (<25 kg/m^2^), overweight (25–30 kg/m^2^), or obese (>30 kg/m^2^). The proportion of underweight participants was very low (below 1%) and these were included in the <25 kg/m^2^ category. Current *smoking* was categorized as yes vs no.

*Leisure-time physical activity* (LTPA) was assessed by eliciting the respondents’ average weekly hours of physical activity during leisure time and commuting over the past 12 months. It was divided into intensity grades as follows: walking (=moderate), brisk walking (=moderate), jogging (=vigorous), and running (=vigorous) or equivalent activities. Each intensity grade question had response alternatives from 0 to at least 4 h/week. We used metabolic equivalents (MET) to approximate the amount of physical activity. We calculated MET hours/week by multiplying the weekly time with the MET value of each intensity and summing these values. Three activity groups were formed: low (under 14 MET hours/week), moderately active (at least 14 MET hours/week of moderate intensity activity), and vigorously active (at least 14 MET hours/week, including vigorous activity).

*Alcohol intake* was assessed by asking ‘which of the following options best describes your current use of beer, wine, and spirits?’, with the following response options (I do not drink alcohol, once a year or less often, 3–4 times a year, about once every couple of months, about once a month, a couple of times a month, once a week, a couple of times a week, 3–4 times a week, daily or almost daily). For this analysis, we categorized the participants’ alcohol consumption into three groups: no (I do not drink alcohol), light to moderate (once a year or less often, 3–4 times a year, about once every couple of months, about once a month, and a couple of times a month), and high (once a week, a couple of times a week, 3–4 times a week, and daily or almost daily).

The covariates were selected as they have been associated with the family-related factors, quality of life or work–family reconciliation.

### Statistical analyses

We investigated the trajectories of WFS using the dichotomous outcome from all four timepoints. Person-centred group-based trajectory analysis using Growth mixture modelling (GMM) was used to model the outcome and to identify the trajectories. First, we analysed the quantity and types of trajectories. We used model fit criteria, including the Akaike Information Criterion (AIC), the Bayesian Information Criterion (BIC), sample size-adjusted BIC, entropy, and posterior probability of trajectory membership (Table S1), to choose the final model [25]. A better model fit is indicated by lower BIC, AIC, and sample size adjusted BIC values, as well as by entropy values near to one, and higher posterior probability. The model's interpretability was also considered. A two-trajectory model was chosen based on the above-mentioned criteria. Plotting the probability against each survey year enabled us to visualize the trajectory groups for both genders.

The characteristics of the study participants are presented as frequencies and percentages by WFS trajectory stratified by gender.

Next, the associations between the family-related, quality-of-life, sociodemographic, and lifestyle variables and the WFS trajectories were studied using log-binomial regression models. We (also) calculated ORs and their 95% CIs. We fit three models separately for women and men: Model I was a crude model, in Model II family-related variables were adjusted for sociodemographic and lifestyle factors (age, gender, and socioeconomic position, current smoking, alcohol intake, BMI, and LTPA), and Model III was simultaneously adjusted for all the studied variables from Model I except LTPA, as this correlated with physical functioning.

GMM was conducted in Mplus v8.2 and all the other analyses in SAS 9.4.

## Results

### WFS trajectories

**Figure 1.**
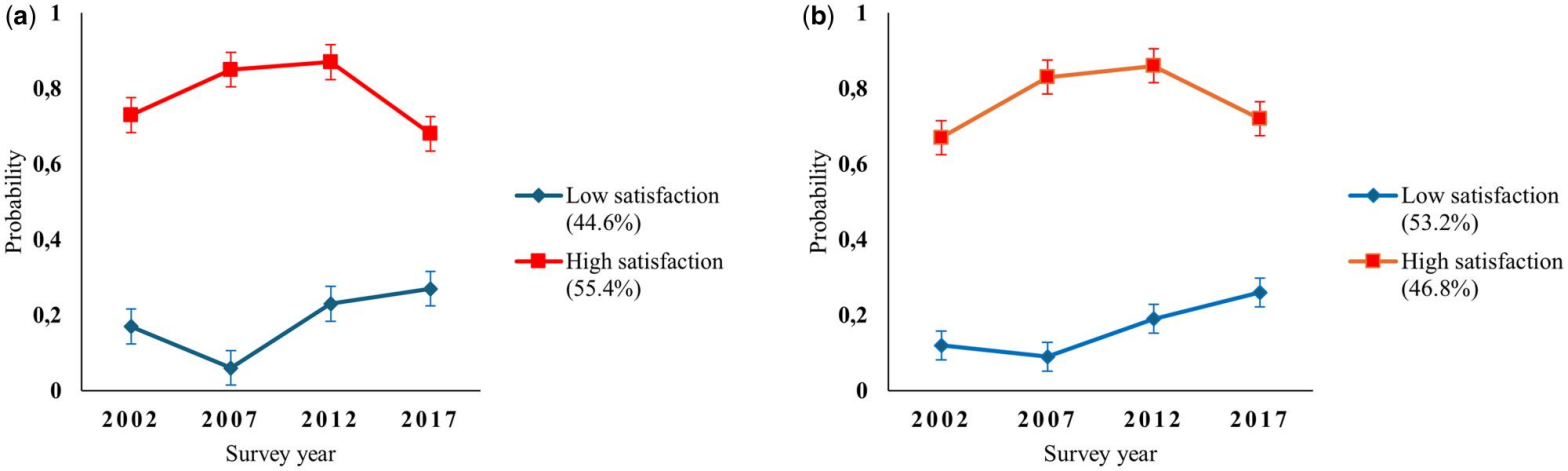
Fifteen-year developmental pathways of work-family conflict among midlife municipal employees among (a) women and (b) men.

We compared the fit statistics as well as the interpretability and meaningfulness of the trajectory model and selected a two-class solution. Of the women, 45%, and of the men, 53% belonged to the low WFS trajectory (Figure 1ab). The proportion of women was thus higher in the high WFS trajectory than in the low (86% vs. 82%). The difference between the two trajectory groups increased first and then diminished during follow-up, with the share of dissatisfied employees increasing and satisfied employees decreasing towards the end of the follow-up among both women and men.

### Respondent’s characteristics by WFS trajectory

The great majority of the respondents were 40 or 45 years old at baseline in both trajectory groups in both genders. We found differences by trajectory in the distributions of respondents in terms of socioeconomic position, and emotional well-being in both genders (Table 1). Among women, the difference was found for having children aged 0–6 and for physical functioning, whereas among men the difference was found for having children aged 7-18 only.

**Table 1.** Baseline characteristics of the studied population by trajectory of satisfaction with work–family reconciliation (WFS), by gender.

Characteristics	Women (*N* = 1416)			Men (*N* = 265)		
	Low WFS	High WFS	*P*-value	Low WFS	High WFS	*P*-value
	(*n* = 631)	(*n* = 785)		(*n* = 141)	(*n* = 124)	
**Age (years)**			.472			.248
40	309 (48.97)	382 (48.66)		70 (49.65)	51 (41.13)	
45	292 (46.28)	354 (45.10)		58 (41.13)	55 (44.35)	
50	30 (4.75)	49 (6.24)		13 (9.22)	18 (14.52)	
**Socioeconomic position**			.005			.036
High	368 (58.97)	401 (51.48)		89 (64.49)	94 (76.42)	
Low	256 (41.03)	378 (48.52)		49 (35.51)	29 (23.58)	
**Currently smoking**			.709			.808
No	489 (77.87)	613 (78.69)		111 (79.29)	99 (80.49)	
Yes	139 (22.13)	166 (21.61)		29 (20.71)	24 (19.51)	
**Alcohol intake**			.767			.980
No	26 (4.19)	38 (4.90)		5 (3.60)	5 (4.07)	
Moderate	326 (52.50)	411 (53.03)		53 (38.13)	47 (38.21)	
High	269 (43.32)	326 (42.06)		81 (58.27)	71 (57.72)	
**BMI (kg/m^2^)**			.500			.645
<25.0	400 (63.39)	515 (65.61)		73 (51.77)	63 (50.81)	
25–29.9	162 (25.67)	198 (25.22)		55 (39.01)	53 (42.74)	
≥30	69 (10.94)	72 (9.17)		13 (9.22)	8 (6.45)	
**Leisure-time physical activity**			.637			.252
Low	127 (20.13)	169 (21.53)		30 (21.28)	29 (23.39)	
Moderate	248 (39.30)	316 (40.25)		37 (26.24)	22 (17.74)	
Vigorous	256 (40.57)	300 (38.22)		74 (52.48)	73 (58.87)	
*Family-related factors*						
**Children aged 0–6**			.004			.583
No	275 (43.58)	403 (51.34)		65 (46.10)	53 (42.74)	
One or more	356 (56.42)	382 (48.66)		76 (53.90)	71 (57.26)	
**Children aged 7–18**			.574			.009
No	124 (19.65)	145 (18.47)		50 (35.46)	26 (20.97)	
One or more	507 (80.35)	640 (81.53)		91 (64.54)	98 (79.03)	
**Spouse lives in same household**			.118			.280
Yes	429 (73.33)	555 (77.08)		96 (72.73)	92 (78.63)	
No	156 (26.67)	165 (22.92)		36 (27.27)	25 (21.37)	
*Quality of life*						
**Physical functioning**			<.001			.493
Low	131 (20.86)	107 (13.65)		15 (10.64)	9 (7.26)	
Moderate	242 (38.54)	251 (32.02)		46 (32.62)	37 (29.84)	
High	255 (40.61)	426 (54.34)		80 (56.74)	78 (62.90)	
**Emotional well-being**			<.001			<.001
Low	278 (44.06)	203 (26.06)		72 (51.43)	34 (27.42)	
Moderate	263 (41.68)	335 (43.00)		53 (37.86)	55 (44.35)	
High	90 (14.26)	241 (30.94)		15 (10.71)	35 (28.23)	

The sample includes those working at the time of all the surveys.

### Associations between family-related factors and WFS trajectories

Table 2 shows the bivariate (Model I) and multivariable (Models II and III) associations of the family characteristics and quality of life related factors with belonging to the low WFS trajectory, adjusted for sociodemographic and lifestyle-related factors, separately for women and men. Having children aged 0–6 years was associated with increased odds of belonging to the low WFS trajectory only among women. This association grew stronger when adjusted for sociodemographic variables in Model II (OR 1.65; 95% CI 1.30–2.10) and remained significant in the final model, Model III (1.52; 1.19–1.95). Having one or more children aged 7–18 was associated with lower odds of belonging to the low WFS trajectory only among men, according to Models II (0.48; 0.24–0.95) and III (0.39; 0.19–0.80).

**Table 2.** Associations of sociodemographic factors, lifestyle, family-related variables, physical functioning, and emotional well-being with the trajectory of low satisfaction with work–family reconciliation among Finnish municipal employees, stratified by gender

Characteristics	OR, 95 % CI					
	Women			Men		
	**Model I** ^a^	**Model II** ^b^	**Model III** ^c^	**Model I** ^a^	**Model II** ^b^	**Model III** ^c^
**Age (years)**						
40	1	1	1	1	1	1
45	1.02 (0.82–1.27)	1.11 (0.88–1.40)	1.08 (0.85–1.37)	0.77 (0.46–1.29)	0.90 (0.50–1.63)	1.03 (0.56–1.90)
50	0.73 (0.49–1.10)	0.76 (0.45–1.29)	0.75 (0.44–1.30)	0.53 (0.24–1.17)	0.62 (0.26–1.48)	0.56 (0.22–1.39)
**Socioeconomic position**						
High	1	1	1	1	1	1
Low	0.74 (0.60–0.91)	0.66 (0.52–0.84)	0.65 (0.51–0.84)	1.78 (1.04–3.07)	1.71 (0.89–3.25)	2.04 (1.04–3.97)
**Currently smoking**						
Yes	1	1	1	1	1	1
No	0.95 (0.74–1.23)	0.89 (0.67–1.18)	0.97 (0.72–1.30)	0.93 (0.51–1.70)	1.17 (0.58–2.36)	1.11 (0.53–2.34)
**Alcohol intake**						
No	1	1	1	1	1	1
Moderate	1.16 (0.69–1.95)	1.11 (0.64–1.93)	1.14 (0.65–2.02)	1.13 (0.31–4.14)	0.73 (0.17–3.13)	0.67 (0.15–2.91)
High	1.21 (0.71–2.03)	1.06 (0.61–1.86)	1.11 (0.62–1.98)	1.14 (0.32–4.10)	0.80 (0.19–3.33)	0.71 (0.17–3.02)
**BMI (kg/m^2^)**						
<25.0	1	1	1	1	1	1
25–29.9	1.05 (0.82–1.35)	1.15 (0.88–1.51)	1.07 (0.81–1.42)	0.90 (0.54–1.49)	0.88 (0.50–1.53)	0.70 (0.38–1.28)
≥30	1.23 (0.87–1.76)	1.43 (0.97–2.11)	1.19 (0.79–1.78)	1.40 (0.55–3.60)	1.42 (0.51–3.95)	1.08 (0.35–3.34)
**Leisure-time physical activity**						
Low	1	1		1	1	
Moderate	1.04 (0.79–1.38)	1.14 (0.84–1.55)		1.63 (0.78–3.38)	1.52 (0.68–3.40)	
Vigorous	1.14 (0.85–1.51)	1.17 (0.86–1.60)		0.98 (0.54–1.79)	0.97 (0.49–1.89)	
**Family-related factors**						
**Children aged 0–6**						
No	1	1	1	1	1	1
One or more	1.37 (1.11–1.69)	1.65 (1.30–2.10)	1.52 (1.19–1.95)	0.87 (0.54–1.42)	1.08 (0.60–1.94)	1.10 (0.60–2.02)
**Children aged 7–18**						
No	1	1	1	1	1	1
One or more	0.93 (0.71–1.21)	0.86 (0.64–1.16)	0.83 (0.61–1.12)	0.48 (0.28–0.84)	0.48 (0.24–0.95)	0.39 (0.19–0.80)
**Spouse living in same household**						
Yes	1	1	1	1	1	1
No	1.22 (0.95–1.57)	1.24 (0.94–1.64)	1.17 (0.88–1.56)	1.38 (0.77–2.47)	1.04 (0.53–2.01)	0.83 (0.41–1.67)
**Quality of life**						
**Physical functioning**						
Low	1		1	1		1
Moderate	0.79 (0.58–1.07)		0.93 (0.67–1.31)	0.74 (0.29–1.89)		0.61 (0.21–1.74)
High	0.49 (0.36–0.66)		0.59 (0.42–0.83)	0.62 (0.25–1.49)		0.76 (0.27–2.14)
**Emotional well-being**						
Low	1		1	1		1
Moderate	0.57 (0.45–0.73)		0.66 (0.51–0.86)	0.46 (0.26–0.79)		0.43 (0.22–0.82)
High	0.27 (0.20–0.37)		0.32 (0.23–0.45)	0.20 (0.10–0.42)		0.20 (0.09–0.46)

Odds ratios (OR) and their 95% confidence intervals (CIs) from log-binomial regression models.

a Model I: Bivariate model.

b Model II: Family-related variables adjusted for sociodemographic and lifestyle factors.

c Model III: Multivariable model, simultaneously adjusted for all variables included in Model I without LTPA as this is correlated with physical functioning.

High physical functioning showed lower odds of belonging to the low WFS trajectory only among women. This remained significant in the final model (0.59, 0.42–0.83). Similarly, compared to low emotional well-being, moderate (OR from Model III 0.63; 0.50–0.80) and high (0.30; 0.22–0.41) emotional well-being was inversely associated with the low WFS trajectory in both women and men (OR for moderate from Model III 0.43; 0.22–0.82, and OR for high 0.20; 0.09–0.46).

Among women, those in a low socioeconomic position had 35% lower odds of belonging to the low WFS trajectory than those in a higher socioeconomic position (final model 0.65; 0.51–0.84), whereas men had a 2- fold higher odds of belonging to the low WFS trajectory (in the final model (2.04, 1.04-3.97).

## Discussion

We studied the 15-year developmental patterns of satisfaction with work–family reconciliation (WFS) and their relationship with family-related factors and quality of life among midlife Finnish municipal employees. We found that about a half of the participants (45% of the women and 53% of the men) belonged to a low WFS trajectory.

In the low WFS trajectory, satisfaction decreased during the first follow-up and then started to increase from the second follow-up onwards, whereas in the high WFS trajectory, it slightly increased during the first follow-up and then started decreasing both among women and men. The probabilities showed clearly differentiated pathways, as the high trajectory remained above 0.70 and the low trajectory below 0.30 throughout the follow-up for both genders.

The membership in the low WFS trajectory closely associated with quality-of-life measures and with having younger vs. older children at home. More specifically, we found that having one or more children aged 0–6 years at home was associated with increased odds of belonging to the low WFS trajectory among women, while having one or more children aged 7–18 was associated with lower odds among men. High physical functioning and high emotional well-being were inversely associated with belonging to the low WFS trajectory among women, while only high-emotional well-being was thus associated among men. We further found that men in a low socioeconomic position had 2-fold higher odds of belonging to the low WFS trajectory than in a higher socioeconomic position. Contrarywise, among women those in a lower socioeconomic position had a 35% lower likelihood of belonging to the low WFS trajectory.

Although our results are consistent with previous findings regarding the developmental pathways of WFS [14, 26], they also differ in many ways. First, we found no earlier studies on the trajectories of the work–family interface that had used a single-item measure. Second, no earlier studies seem to have studied the associations of these trajectories with family-related characteristics. Nonetheless, some earlier studies based on multi-item work-to-family and family-to-work measures have reported trajectories varying in number, from three to twelve [14, 26].

A few earlier studies [26, 27] described 6–12 trajectories for men and women separately. The men in these studies frequently belonged to fewer trajectories than the women. Stafford *et al*. [27] identified eight trajectory types among both men and women, but 50% of the men, as opposed to 31% of the women, belonged to the one most prevalent trajectory type. In addition, women's work–family trajectories were discovered to be more complex, with more transitions between various work and family states, meaning that women's internal variety was higher than that of men [28].

Our results among women regarding younger children are in line with earlier findings showing that participants who had to take care of children at home reported more work–life stress than those who had no care responsibilities [29]. This is likely because more time and effort is usually needed to take care of very young children than older ones, which can affect the working schedule of one or both parents. As children grow and become more independent, parents are better able to focus on their work or recreational activities, which in turn increases life satisfaction. In our study, those having children aged 7–18 years at home had a lower likelihood of belonging to the low WFS trajectory, but this was statistically significant only among men. Our findings imply that women take more responsibility of care of their younger children than men, while satisfaction in work-family reconciliation especially among men increases when children have become more independent of their parents. The previous evidence on the effect of gender on WFS is mixed. Some studies have found no gender effect on WFS [30] or higher satisfaction among men [18].

Of the quality-of-life measures, particularly emotional well-being was strongly inversely associated with the low WFS trajectory among both genders, while the association with physical functioning was statistically significant only among the larger group of women. According to our knowledge, this relationship has not been studied previously, although some earlier findings have shown a strong correlation between the work–family interface and quality of work life [31]. Research suggests that the pressure caused by incompatible responsibilities reduces well-being in the work domain more than in the non-work domain, regardless of the cause of WFC [31]. Few studies have directly measured physical health, while many have found some indicators of mental health or psychological stress, such as depressive symptoms, emotional strain, distress, burnout, or reduced life satisfaction, to be linked with higher levels of dissatisfaction with work–family reconciliation [8, 32]. The effort–recovery model [33] assumes that putting much effort into both the job and personal life can lead to several physiological and behavioural processes that are detrimental to health. The negative effect of work-related effort can be reduced by activities that speed up recovery, but these processes can also be reversed. That is, stress is released during the healing process, which leads to the restoration of health and well-being [34]. By lowering the detrimental impacts of workload, one can expect a long-term beneficial effect on health as resources are replenished and moods elevated [35].

Of the lifestyle factors, LTPA, alcohol intake and BMI had no significant relationship with low WFS. Earlier studies have suggested that LTPA is particularly effective in reducing stress and enhancing health, although many other leisure activities, such as traveling, playing video games, or volunteering, are also known to restore positive resources [33, 34].

Among the sociodemographic factors, Socioeconomic position was differently associated with WFS trajectories in women and men: in men low socioeconomic position doubled the risk of belonging to the low WFS trajectory, while in women it was protective of it. The finding among men is in line with previous findings that economic inequality mainly affects participants in the lower strata [36]. Low income was associated with a higher work-life conflict among men in an earlier study [37]. In another study work-life balance showed stronger associations with mental health for individuals with high socioeconomic position both in women and men [38].

Keeney *et al*. [38] have identified non-work domains of relevance for the work–life balance in the quality-of-life literature [39], the Multiple identity perspective [40], and in Super's [19] Life-space theory of career development. These domains are *education, health, leisure, friendships, romantic relationships, family, household management,* and *community involvement*. However, many of these domains had different levels of importance for different individuals [39]. Moreover, if an individual's interests and circumstances vary, the relative relevance of these life domains is likely to shift [19]. Therefore, it is critical to comprehend whether other non-work domains are equally vital to family and the point at which priorities shift.

### Strengths and limitations

The results were based on the representative data of municipal workers in the City of Helsinki, which is probably comparable to municipal employees in any Nordic welfare state. The response rates in Phase 1 and over the follow-ups were high. We studied only those who were aged between 40 and 50 in the Phase 1 survey and who were working either full-time or part-time during Phases 2–4 surveys. This effectively captured the work-relatedness of WFS as well as work-related exposures. Our outcome variable was based on a single-item measure, unlike in many previous studies. A single-item measure is more practical to use, and this measure seemed to capture all the aspects of work–family interface issues. It is also a validated tool [16]. The data enabled us to account for factors from several important domains that might obscure the analysed associations, and the study’s prospective design allowed us to examine the developmental paths, which reduces the likelihood of inverse causation. Self-reported measures are susceptible to potential reporting bias. Furthermore, we cannot rule out the healthy worker effect, as occupational cohorts tend to exhibit better health than the general population. A relatively large number of participants who responded to the Phase 1 survey were excluded on the basis of our inclusion criteria, but the data have been deemed representative of the target population [22]. Younger age, a lower occupational class, and sickness absences were associated with non-response [22].

## Conclusions

In conclusion, a follow-up of over 15–17 years of an occupational cohort of employees of the City of Helsinki showed that more than half of the women employees belonged to the high WFS trajectory, while less than half of the men employees had high satisfaction in WFS trajectory. High quality of life was associated with a low likelihood of low satisfaction, having underaged children was associated with a high likelihood of low satisfaction among women only and having children aged 7–18 years was associated with a lower likelihood of low satisfaction among men only. Our findings offer guidance for future research on mental stress at work and its effect on the work–family life balance as well as its consequences on the future health of employees. Solutions to these issues should be actively sought at the workplace.

## Supplementary Material

ckae117_Supplementary_Data

## Data Availability

Dataset cannot be shared publicly as it contains confidential medical information. The study participants as well as the City of Helsinki have not provided their permission to data share. The data are available upon agreement with the Helsinki Health Study for researchers who meet the criteria for access to confidential data. Researchers interested in the data may contact the Helsinki Health Study group (email: kttl-hhs@helsinki.fi). Key points Two distinct trajectories of satisfaction with work–family reconciliation (WFS) were identified over the follow-up of over 15–17 years of an occupational cohort, with about a half of the participants (45% of the women and 53% of the men) belonging to the low WFS trajectory. Having underaged children was associated with a high likelihood of low satisfaction among women only and having children aged 7–18 years was associated with a lower likelihood of low satisfaction among men only. High quality of life, particularly emotional well-being was associated with a low likelihood of low satisfaction.
