# Supplementary material for: Trajectories of satisfaction with work–family reconciliation among midlife employees: the role of family-related factors and quality of life
Source: Eur J Public Health. 2024 Jul 25;34(6):1059–65. doi: 10.1093/eurpub/ckae117 (PMC11631479; doi:10.1093/eurpub/ckae117)
Supplement: ckae117_Supplementary_Data [file ckae117_supplementary_data.pdf]

Supplementary Table S1: Fit criteria for model selection in total sample.

Class	BIC	AIC	Sample size adjusted BIC	Entropy	Group proportion	Posterior probability	Lo-Mendell-Rubin P-value
**2**	**28116.8**	**28067.4**	**28094.5**	**0.461**	**45/55**	**0.82/0.85**	**<0.001**
3	28102.9	28025.3	28067.9	0.418	45/18/37	0.82/0.64/0.52	0.012
4	28115.2	28009.4	28067.5	0.468	38/40/16/6	0.53/0.85/0.65/0.10	<0.001
5	28149.7	28015.6	28089.3	0.475	22/47/9/3/19	0.78/0.78/0.29/0/0.21	0.545

Note: The two-class model was chosen as the best fitting model. Although the lowest BIC and AIC values were found in the three- and four-class models, and the highest entropy in the five-class model, some posterior probabilities were quite low for the three- to five- class models.
